# Comprehensive Assessment of Anti-Inflammatory, Antiproliferative and Neuroprotective Properties of Cauliflower after Dehydration by Different Drying Methods

**DOI:** 10.3390/foods13193162

**Published:** 2024-10-04

**Authors:** Antonio Vega-Galvez, Alexis Pasten, Elsa Uribe, Nicol Mejias, Michael Araya, René L. Vidal, Gabriela Valenzuela-Barra, Carla Delporte

**Affiliations:** 1 Food Engineering Department, Universidad de La Serena, Av. Raúl Bitrán 1305, La Serena 1700000, Chile; afpasten@userena.cl (A.P.); muribe@userena.cl (E.U.); susana.mejias@userena.cl (N.M.); 2 Instituto Multidisciplinario de Investigación y Postgrado, Universidad de La Serena, Av. Raúl Bitrán 1305, La Serena 1700000, Chile; 3 Centro de Investigación y Desarrollo Tecnológico en Algas (CIDTA), Facultad de Ciencias del Mar, Universidad Católica del Norte, Larrondo 1281, Coquimbo 1780000, Chile; mmaraya@ucn.cl; 4 Facultad de Medicina, Instituto de Neurociencia Biomédica (BNI), Universidad de Chile, Santiago 8380000, Chile; renevidalg@gmail.com; 5 Centro FONDAP de Gerociencia, Salud Mental y Metabolismo (GERO), Santiago 8380000, Chile; 6 Centro de Biología Integrativa, Facultad de Ciencias, Universidad Mayor, Santiago 8380000, Chile; 7 Laboratorio de Productos Naturales, Facultad de Ciencias Químicas y Farmacéuticas, Universidad de Chile, Santiago 8380000, Chile; gabriela.m.valenzuela@ciq.uchile.cl (G.V.-B.); cdelpor@uchile.cl (C.D.)

**Keywords:** *Brassica oleraceae* L. var. Botrytis Linnaeus, cell lines, drying methods, phlogistic agents

## Abstract

Cauliflower (*Brassica oleraceae* L. var. Botrytis Linnaeus) has various health benefits due to its rich bioactive compound content. However, this fresh vegetable faces challenges related to its perishability and short shelf life. This study explores the effect of five drying methods, namely vacuum drying (VD), convective drying (CD), infrared drying (IRD), low-temperature vacuum drying (LTVD) and vacuum freeze-drying (VFD), on the bioactive compounds and health-promoting properties of cauliflower. Analyses of amino acids, hydroxycinnamic acid and its derivatives, glucosinolates, and isothiocyanates, as well as evaluations of their anti-inflammatory, antiproliferative, and neuroprotective properties, were conducted based on these five drying methods. The results revealed that samples treated with VFD and IRD had a higher content of amino acids involved in GSL anabolism. Moreover, VFD samples retained hydroxycinnamic acid derivatives and glucosinolates to a greater extent than other methods. Nonetheless, the CD and VD samples exhibited higher antiproliferative and neuroprotective effects, which were correlated with their high sulforaphane content. Overall, considering the retention of most bioactive compounds from cauliflower and the topical inflammation amelioration induced in mice, VFD emerges as a more satisfactory option.

## 1. Introduction

Dietary intake of cruciferous vegetables is one of the most important natural chemopreventive strategies, particularly due to their role in reducing inflammation [1,2]. The chemopreventive effect of cruciferous vegetables can be attributed indirectly to the presence of glucosinolates (GSLs), which are naturally found in almost all these vegetables [3,4]. Specifically, cauliflower (*Brassica oleraceae* L. var. Botrytis Linnaeus) contains abundant GSLs, mainly glucobrassicin, which is an indolyl GSL [5,6,7]. Other GSLs include glucoraphanin, sinigrin, gluconapin, progoitrin, neoglucobrassicin and 4-methoxy-glucobrassicin [6,8,9,10,11]. Importantly, GSLs are not bioactive per se until they have been hydrolyzed by an enzyme called myrosinase, which is activated in response to plant tissue damage after pathogen attack or on chopping or grinding during raw material preparation. This hydrolysis produces a range of metabolites including isothiocyanates (ITCs) that are directly responsible for the chemopreventive activity of cruciferous vegetables [3,12,13,14,15,16]. Some experiments conducted on various cell lines and animals suggest that ITCs exert their chemopreventive effects by modulating phase I and phase II detoxification enzymes. This modulation reduces the bioactivation of carcinogens, resulting in less carcinogen binding to DNA and a decrease in pro-mutagenic effects [1,17]. ITCs have also been shown to downregulate the nuclear factor kappa B (NF-κB) signaling pathway indirectly via crosstalk with nuclear factor erythroid 2-related factor 2 (Nrf2) and may therefore reduce inflammation, a well-recognized risk factor in carcinogenesis and neurodegeneration [16,18,19].

To fully benefit from the health-promoting properties of ITCs, it is important that GSLs undergo enzymatic hydrolysis. However, this reaction should ideally take place after the consumption of cruciferous vegetables, rather than before, to maximize the potential health benefits. It is known that the water present in fresh crucifers facilitates the enzymatic hydrolysis of GSLs [20]. Although this problem can be solved by properly controlling the water content by drying operations. For example, in convective drying, heat transfers from the hot air to the cooler food due to the temperature gradient, inducing evaporation. In infrared drying, when the frequency of infrared radiation matches the natural frequency of water molecules within the food, it causes friction and collisions between these molecules, thereby promoting evaporation [21]. In vacuum drying and low-temperature vacuum drying, the rate of evaporation increases because the boiling point of water is reduced as the pressure in the drying chamber decreases. Finally, in vacuum freeze-drying, water is removed through sublimation [22].

It is well known that different drying methods have different effects on the GSL and other components based on the drying condition employed and exposure times [15]. Tetteh et al. [23] found that oven-, sun- and freeze-drying methods can be efficient in drying *Moringa oleifera* leaves, maintaining higher intact GSL contents compared with solar and shade drying. Korus et al. [24] also found that blanched kale leaves previously hot air- or freeze-dried had a significantly higher GSL content than that obtained from unblanched dried leaves. Blanching VFD could also maintain a high content of GSL and ITC in some Brassicaceae vegetables during transport and storage [15]. Ferreira et al. [25] observed that VFD had no significant impact on the GSL content in broccoli, whereas a blanching process followed by air-drying resulted in a reduction of GSL by more than 50%.

Although numerous studies on drying fruits and vegetables have been conducted in recent years, research on drying cauliflower remains scarce. This study assessed the effect of five drying methods, namely vacuum drying (VD), convective drying (CD), infrared drying (IRD), low-temperature vacuum drying (LTVD) and vacuum freeze-drying (VFD), on the bioactive compounds and health-promoting properties of cauliflower. Analyses of amino acids, hydroxycinnamic acid and its derivatives, GSLs, and ITCs, as well as evaluations of their anti-inflammatory, antiproliferative, and neuroprotective properties, were conducted based on these five drying methods, aiming to preserve the bioactive compounds for their application as functional ingredients.

## 2. Materials and Methods

### 2.1. Raw Material and Drying Procedure

Cauliflower (*Brassica oleracea* var. botrytis Linnaeus) was sourced from a local market, Coquimbo Region, Chile. The cauliflower heads were cut into small florets after removing the leaves and stems. The florets were then blanched by submerging them in boiling water for 30 s to deactivate the epithiospecific protein and rapidly cooling them in an ice-water mixture. After cooling, the florets were centrifuged in a vegetable centrifuge to remove excess water. A well-mixed batch of the florets (2 kg) was then divided into five equal portions for drying by the following methods: (i) VD (Memmert, model VO 400, Schwabach, Germany) at a temperature of 60 °C with a vacuum pressure of 10 kPa; the drying time was 12 h, and the final water content was 11.65 g/100 g; (ii) CD, performed using a convective system at 60 °C with a constant air flow rate of 1.5 ± 0.2 m/s; the drying time was 8 h, resulting in a final moisture content of 9.05 g/100 g; (iii) IRD, carried out using a system equipped with two 175 W infrared emitters at 60 °C for 8 h; the final water content was 10.31 g/100 g; (iv) LTVD (Memmert, model VOcool 400, Schwabach, Germany), carried out at a temperature of 20 °C and a vacuum pressure of 1 kPa; tdrying time was 36 h, and the final water content was 12.87 g/100 g; and (v) VFD (AdVantage Plus, Gardiner, NY, USA) with a condenser temperature of −60 °C and a vacuum pressure of 0.027 kPa; the drying time was 12 h, resulting in a final moisture content of 8.88 g/100 g. The selection of drying methods used in this study was based on the methods employed in our previous research [22]. After the drying processes were completed, the samples were ground into powder using a basic analytical mill (IKA A-11 basic, Wilmington, DE, USA) and sieved through a 1.00 mm mesh sieve. All samples were stored separately in sealed plastic bags.

### 2.2. Determination of Amino Acid Composition

The amino acid profile was analyzed using high-performance liquid chromatography (HPLC) with pre-column derivatization [26]. Cauliflower powder (200 mg) was placed in a hydrolysis tube with 10 mL of 6 M HCl, then autoclaved for 2 h at 120 °C. After hydrolysis, the solution was diluted with ultra-pure water to 50 mL. A 100 μL aliquot was adjusted to pH 10 with borate buffer, evaporated to dryness, and reconstituted to 200 μL with the same buffer. Then, the reconstituted solution was filtered using a 0.22 μm Nylon syringe filter. Standard casein was treated in the same way as the sample after hydrolysis.

The pre-column derivatization was performed using the Jasco AS-2055 auto sampler as described by Araya et al. [27]. Briefly, 20 μL of amino acid standard (AAS-18, Sigma Aldrich, St. Louis, MO, USA) mix was combined with 10 μL of each external standard (glutamine, asparagine, and tryptophan) and 10 μL of borate buffer (pH 10.2). In step 1, 20 μL of the standard mix or hydrolyzed sample was mixed with 30 μL of borate buffer. In step 2, the mixture was treated with air and added to 20 μL of o-phthalaldehyde (OPA). In step 3, the final mixture was placed in a vial and homogenized with air before being injected into the equipment. Three mobile phases were used for detection: (A) borate buffer pH 7.8; (B) acetonitrile: methanol: water (90:90:10), and (C) 100% methanol. The flow rate was 2.0 mL/min. The ramp begins at 0–1.9 min, 100% (A); 18.1–18.6 min, 42% (A) 58% (B); 22.3 min, 30% (A) 70% (B); 22.40–26.00 min, 100% (C); 26.10–28.00 min, 100% (A). Separation was conducted using an Agilent ZORBAX Eclipse AAA reversed-phase column (150 × 4.6 mm, 3.5 μm; Santa Clara, CA, USA) maintained at a temperature of 40 °C. The injection volume was 10 μL, and the chromatographic run time was 28 min. Detection involved capturing spectra between 240 nm and 400 nm, with measurements taken specifically at 338 nm.

### 2.3. Determination of Glucosinolate Content and Hydroxycinnamic Acid and Its Derivatives

For extraction of GSLs and hydroxycinnamic acid and its derivatives, each sample powder (200 mg) was mixed with 990 µL of 80 % (*v*/*v*) methanol, with 10 µL of 1000 µg/mL salicylic acid added as an internal standard to the extraction solvent. The mixture was stirred in a cell disruptor three times for 3 min each. Next, the mixture was centrifuged at 12,000× *g* rpm for 15 min. The supernatant was filtered through a 0.22 µm PTFE membrane, and 200 µL of it was evaporated to dryness with nitrogen using a BIOBASE sample concentrator MD200-1 (Sysmatec, Eyholz, Canton of Valais, Switzerland). Then, the remaining residue was resuspended in 50 µL of the extraction solution and transferred to a vial for the chromatographic analysis. The analysis was conducted with a Dionex Ultimate 3000 UHPLC system (Thermo Fisher Scientific, Sunnyvale, CA, USA). A reversed-phase HPLC column, Kinetex^®^ C18 (50 mm × 2.1 mm; 2.6 µm), from Phenomenex (Torrance, CA, USA), was employed. The mobile phase was used in gradient mode as follows: 80% of eluent A (100% water containing 48 mM formic acid) and 20% of eluent B (50% acetonitrile:50% water) were maintained with 48 mM formic acid for 1.0 min, followed by a linear increase to 100% B in 8.0 min and holding for 5 min. Afterward, eluent C (methanol) was added for 2 min and, finally, the mixture was equilibrated with the initial conditions for 2 min. The injection volume was 5 µL, and flow rate was set at 0.4 mL/min. Detection was carried out using a high-resolution mass spectrometer Q Exactive Focus with an Orbitrap detector, equipped with an electrospray interface HESI II (Thermo Fisher Scientific, Sunnyvale, CA, USA), and quantification was by the internal standard method using salicylic acid according to Vega-Galvez et al. [28]. The identification of the compounds was facilitated by using the SIRIUS 5.7.0. software and the PubChem database.

### 2.4. Determination of Isothiocyanate Content

For extraction of ITCs, 5 g of cauliflower powder was mixed with 40 mL of Milli-Q water and incubated at 37 °C with agitation at 200 rpm for 3 h. The sample was then transferred to a separating funnel containing 10 mL of dichloromethane, shaken gently, and the organic layer was collected. This procedure was performed twice. The solid residue was gathered, centrifuged for 5 min at 10,000× *g* rpm, and the organic phase was collected, evaporated, and reconstituted with 1 mL of acetonitrile. After filtration of the reconstituted organic phase through 0.22 µm cellulose acetate filters, extracts were analyzed via a HPLC Waters LC system (Waters, Milford, MA, USA) equipped with a Waters^®^ 2996 photo Diode Array Detector (DAD). Chromatographic separation was carried out with a 2.5 μm, 4.0 × 250 mm Spherisorb^®^ ODS-2 column (Waters, Milford, MA, USA), which was set at 25 °C. The mobile phases used were (A) 0.05% phosphoric acid in acetonitrile/water (5:95) and (B) 0.05% phosphoric acid in acetonitrile/water (95:5). The flow rate was fixed at 1 mL/min, and the injection volume was 20 µL [29]. Chromatographic results were collected using Empore 3.0 software (Waters, Milford, MA, USA). The individual ITCs were identified and quantified in comparison with the retention time and external standard curve of four ITC standards (sulforaphane [SFN], allyl isothiocyanate [AITC], phenyl isothiocyanate [PITC] and indole-3-carbinol [I3C]), and were expressed in microgram per gram (μg/g) of dried sample.

### 2.5. Extraction Procedure for Determining Anti-Inflammatory, Antiproliferative, and Neuroprotective Properties

Two grams of cauliflower powder was mixed with 10 mL of 80% methanol and stirred for 1 h, followed by centrifugation at 4193× *g* for 20 min at 4 °C. The supernatant was filtered using a Whatman #1 filter. Simultaneously, the residue was re-extracted twice using the same procedure, and all three filtrates were combined and evaporated under vacuum using a Multivapor evaporator at 40 °C. The resultant extract was lyophilized (solid extract) and then reconstituted in acetone for anti-inflammatory analysis, in RPMI medium for antiproliferative analysis and in dimethyl sulfoxide (DMSO) for neuroprotective analysis.

### 2.6. Determination of In Vivo Anti-Inflammatory Potential

The animal assays were conducted as reported earlier [30] with some modification. Firstly, BALB/c juvenile mice (20–25 g) obtained from the stock at the Bioterium of Faculty of Medicine, University of Chile were used. Subsequently, inflammation was induced in the right ear in the control mouse group (*n* = 16) by the topical application of 5 μg/ear of phorbol 12-myristate 13-acetate (TPA) or 2 mg of arachidonic acid (AA) dissolved in 20 μL of acetone (negative control = 100% inflammation), while acetone (20 μL) was topically applied to each mouse’s left ear (vehicle). In parallel, extracts reconstituted in acetone (3.0 mg/ear) obtained from dried cauliflower by different methods or indomethacin (1.0 mg/ear; positive control or reference drugs against TPA) or nimesulide (0.5 mg/ear; positive control or reference drugs against AA) were applied to the other groups of mice immediately after inducing right ear inflammation (group treated with anti-inflammatory agents; *n* = 8). The left ear in this group also received only acetone as vehicle. After 6 h or 1 h of ear edema induction by TPA or AA, respectively, the mice were euthanized by CO_2_ asphyxiation, and ear samples (6 mm of tissue from both ears) were collected using a mechanical punch. Edema was measured as the difference in the weights between the punches from right and left ears of the negative control and the treated mouse groups, respectively.

### 2.7. Determination of In Vitro Antiproliferative Potential

Both non-tumorigenic gastric GES-1 cell lines and the gastric cancer AGS cell line were grown and maintained in RPMI-1640 medium, supplemented with 10% Fetal Bovine Serum (FBS) and 1% penicillin-streptomycin, inside an incubator (Memmert, INCO153med, Schwabach, Germany) at 37 °C, 5% CO_2_, and 95% humidity. Then, the cells (5000 cells/well) were seeded in sterile 96-well plates. After a 24-hour period, both cancer cells and non-tumorigenic cells were incubated for 48 h in an incubator with 5% CO_2_ with progressive doses of extracts reconstituted in RPMI medium (1.25, 2.5, 5, 10, 15 and 20 mg/mL) obtained from dried cauliflower by different methods. DMSO 50% and RPMI-1640 medium were used as positive and negative controls, respectively. Using the propidium iodide (PI) technique [22], cell death was measured at emission and excitation wavelengths of 617 and 535 nm, respectively, in a fluorescent microtiter reader (TECAN Infinite M Nano+, Maennedorf, Switzerland). Non-linear regression was used to calculate the dose required to reduce the number of viable cells by half (IC50).

### 2.8. Determination of In Vitro Neuroprotective Potential

Mouse dopaminergic-derived SN4741 neuronal cells (American Type Culture Collection, Manassas, VA, USA) were cultured in Dulbecco’s Modified Eagle Medium (DMEM) containing 10% fetal bovine serum (Life Technologies, Thermo Fisher Scientific, Waltham, MA, USA) and 1% penicillin-streptomycin. The cultures were maintained in an incubator with 5% CO_2_ at 37 °C. The cells were then seeded in a 96-well plate at a density of 1 × 10^4^ cells/mL and treated with various concentrations of extracts reconstituted in DMSO obtained from cauliflower dried by different methods (10, 50, and 100 μg/mL). The negative control group was treated with DMSO alone, while the positive control group was treated with α-synuclein (α-syn). After 24 h, the cytotoxicity of the extracts was determined by measuring intracellular Sytox green levels. Once cytotoxicity was evaluated, SN4741 cells were treated with cauliflower extracts at the appropriate concentration and exposed to human α-synuclein monomers (Mono) or α-synuclein preformed fibrils (α-syn PFF) to assess the neuroprotective ability of the extracts, as previously described [28,31].

### 2.9. Data Analysis

The results were statistically compared according to the distribution of the data, applying appropriate tests depending on whether they followed a normal or non-normal distribution. Where it was pertinent, the data are presented as mean ± SD or mean ± SEM. The Shapiro–Wilk test was used to assess whether the data were normally distributed. For data that exhibited a normal distribution, a one-way ANOVA was applied, followed by post hoc multiple comparison tests (Duncan’s test) to determine which specific group means were significantly different from each other. For data that did not follow a normal distribution, the Kruskal–Wallis non-parametric test was applied, followed by individual comparisons using post hoc tests (Mann–Whitney test) to determine differences between two independent groups. A *p* value of <0.05 was considered significant (* *p* < 0.05, ** *p* < 0.01, **** *p* < 0.0001). The software employed for statistical analysis was Statgraphics Centurion Version 18.1.12 (Statgraphics Technologies, Inc., The Plains, VA, USA). Python version 3.9.4 (Python Software Foundation, Beaverton, OR, USA) was used to create heat map plots that displayed the Pearson correlation between the variables. To conduct this analysis, the necessary libraries such as pandas and seaborn were imported. First, the correlation coefficients were calculated using pandas and stored in a DataFrame. Then, seaborn was utilized to generate the heat map plots, configuring the visualization parameters to highlight significant correlations between the variables.

## 3. Results and Discussion

### 3.1. Amino Acids of Dried Cauliflower

It is noteworthy that amino acids are important primary metabolites in members of the family Brassicaceae because some of them act as precursors for glucosinolate (GSL) biosynthesis. Thus, to gain an insight into the effect of drying on the composition of amino acids in cauliflower, individual amino acids in the dried vegetable obtained through different methods were quantified (Table 1).

Regardless of the drying method used, the following amino acids were found in all samples albeit in different amounts: aspartate, glutamate, serine, glycine, threonine, arginine, alanine, tyrosine, valine, phenylalanine, isoleucine, leucine and lysine, which is consistent with most studies [32,33]. The only difference was found for the case of histidine, which was found in three (IRD, LTVD and VFD) of the five drying methods. As expected, the cauliflower dried by CD had a lower content of total essential amino acids (2.86 g/100 g) compared with the other drying methods. Threonine, isoleucine and lysine were significantly reduced by CD, due to the oxidation of these amino acids by the heat and air or for consumption as substrate in the Maillard reaction, as reported in the literature [34]. Furthermore, it is worth noting that VD, LTVD and VFD operate under vacuum conditions, so they significantly prevent oxidative processes, further protecting the amino acids from degradation. Interestingly, a negative impact of heat application (60 °C) on the total amount of essential amino acids (6.31 g/100 g) was not observed in the IRD sample, being similar to its counterpart obtained by VFD (6.55 g/100 g). This may be due to a reduced necessity of air flow across the product and conversion of radiant energy into heat that may hydrolyze the protein molecules and release certain amino acids [35].

Finally, the data acquired in our study indicate that all amino acids involved in GSL anabolism, including phenylalanine, tyrosine, alanine, valine, leucine and isoleucine, were significantly higher in VFD and IRD compared to other methods, as precursor amino acid content is a crucial factor affecting the synthesis of GSL (Table 1).

### 3.2. Hydroxycinnamic Acid and Its Derivatives in Dried Cauliflower

While outstanding bioactive components of members of the Brassicaceae family are glucosinolates and their hydrolytic products, some other secondary metabolites of interest have also been identified. By liquid chromatography-high-resolution mass spectrometry (LC-HRMS) analysis, we identified hydroxycinnamic acid and some derivatives (mainly of sinapic acid) in cauliflower, which is consistent with previously reported findings [9,36,37,38,39]. As observed in Table 2, the different drying methods had significant effects on the concentration of specific hydroxycinnamic acid and its derivatives.

For example, the amount of chlorogenic acid in LTVD samples was significantly higher than that in samples processed with the other four drying methods (*p* < 0.05). Previous research has highlighted the high temperatures to facilitate the non-enzymatic degradation of chlorogenic acid [40]. However, it can also be seen that the VFD process affects the stability of chlorogenic acid. It is very probably due to the damage to the cell structure caused by the formation of ice crystals during the prior freezing step and subsequent VFD, which increases the activity of the browning enzymes polyphenol oxidase (PPO) and peroxidase (POD) [41]. On the contrary, VFD samples exhibited the highest levels of ferulic acid acyl-β-D-glucoside (*p* < 0.05), possibly caused by the acyltransferase enzymatic activity responsible for the acylation of the ferulic acid glycosides [38,40]. Regarding sinapic acid, with the major quantity in our samples, it was present as various derivatives, such as sinapoyl D-glucoside, 3′-O-sinapoyl-6-O-feruloylsucrose and (3-sinapoyl)fructofuranosyl-(6-sinapoyl)glucopyranoside. Among them, sinapoyl D-glucoside is one of great interest and was the most abundant in dried cauliflower, in a range of 108.6–344.1 µg/g (Table 2), which is within the range reported previously in different Brassica vegetables [39]. As expected, the highest amount of sinapoyl D-glucoside was recorded in the VFD sample, while the lowest was found in the IRD sample. This is not surprising, since some studies carried out by Cong et al. demonstrated that sinapic acid in rapeseed is heat decarboxylated when exposed to a microwave irradiation process, forming another biocompound, canolol [42,43].

### 3.3. Glucosinolate (GSL) Content in Dried Cauliflower

In the current study, GSLs occurring in dried cauliflower were identified in negative ion mode ([M-H]^−^) by LC-HRMS, since only the high resolution and mass accuracy allowed us to distinguish, as deprotonated molecules, neoglucobrassicin (mass-to-charge [*m*/*z*] 447.0648) and 4-methoxy-glucobrassicin (*m*/*z* 447.0688) with the same nominal *m*/*z* (i.e., 447). Other identified GSLs were glucoerucin (*m*/*z* 420.0371) and glucobrassicin (*m*/*z* 447.0556) (Table 2). Among these compounds, glucobrassicin has been the most common GSL identified in cauliflower by other researchers [5,6,7], followed by neoglucobrassicin and 4-methoxy-glucobrassicin [8,9,10,11], while the glucoerucin was present in only trace amounts. The results shown in Table 2 indicate that the VFD method was the most suitable for preserving all detected GSLs in dried cauliflower. Apparently, freezing the samples at −80 °C prior to VFD may have stopped myrosinase activity and preserved the integrity of the tissue, thereby reducing the hydrolysis of GSLs by enzyme [23]. In contrast, the IRD method caused a significant reduction in all detected GSLs (*p* < 0.05). This could be due to the absorption of radiative energy by some components of the sample, which intensifies molecular vibrations, thereby causing intense internal heating and thermal fracture of material structure, which was reported to facilitate the release of GSLs from vacuoles to be rapidly hydrolyzed by myrosinase [44,45]. It is noteworthy to say that the stability of individual GSLs also varied by the drying condition employed. For instance, 4-methoxy-glucobrassicin content was low in cauliflower samples dried by VD and CD at 60 °C, but it was maintained comparatively higher in LTVD samples at 20 °C. Interestingly, the contents of glucobrassicin and neoglucobrassicin were markedly higher in VD and CD samples, respectively, than in those obtained from LTVD. This suggests that the myrosinase activity was partially inactivated by both the employed VD and CD drying conditions [15], resulting in a reduced enzymatic degradation of glucobrassicin and neoglucobrassicin, respectively. However, in LTVD, the temperature (20 °C) was not high enough to deactivate the myrosinase [26], which could explain the relatively high losses of these compounds with this drying method.

### 3.4. Isothiocyanate (ITC) Content in Dried Cauliflower

The nature of the breakdown products of detected GSLs in dried cauliflower was investigated based on calibration curves of authentic standards. As shown in the previous section, glucobrassicin was the major glucosinolate in dried cauliflower. Upon being degraded by myrosinase, glucobrassicin is converted to indole-3-carbinol (I3C). However, this molecule was not detected in any of the dried samples analyzed when comparing their retention times and mass spectra with those of the commercially acquired I3C reference standard. Our findings are consistent with a previous report where indole-3-acetonitrile (I3A), which is another breakdown product of glucobrassicin, was absent in cauliflower treated by γ-irradiation, possibly because the breakdown products of glucobrassicin are more stable at acidic pH [7]. As shown in Table 3, allyl isothiocyanate (AITC) and sulforaphane (SFN) concentrations ranged from 6.00 µg/g to 26.0 µg/g and from 5.40 µg/g to 12.0 µg/g of dried sample, respectively.

The AITC value was within the range of that reported by Cuellar-Nuñez et al. [1] in VFD-treated cauliflower (12.55 µg/g). We could not find previous references to contrast the SFN content of cauliflower (either fresh or dried). It was only found in a study from some years ago in 3-day-old and 9-day-old cauliflower seedlings [46]. It is worth mentioning that AITC is derived from sinigrin, whereas SFN is derived from glucoraphanin. Although both GSLs were not detectable by our analytical method, some studies have been able to detect both sinigrin and glucoraphanin in cauliflower [6,7,8,9,11,15,17]. Under this approach, we speculate that the lowest and highest final losses of glucoraphanin were found in the VFD- and IRD-treated samples, respectively, since the VFD sample had a lower level of SFN (5.40 µg/g) and the IRD sample had a higher content (12.0 µg/g). This result corroborates the fact that IRD can enhance the myrosinase–GSL interaction mechanism and accelerate the conversion process to its ITC analog, whereas the pre-freezing step for VFD minimizes enzyme activity in cauliflower. Unlike SFN, AITC was higher in the LTVD sample, possibly because the temperature regime was not high enough to denature myrosinase. Thus, its activity might have continued to hydrolyze its GSL precursor. In conclusion, drying can induce the conversion of GSLs and promote their hydrolysis to ITCs. However, it seems that this will depend on how the method affects the myrosinase–GSL–ITC interaction mechanism [15].

### 3.5. Anti-Inflammatory Potential in Dried Cauliflower

**Figure 1:**
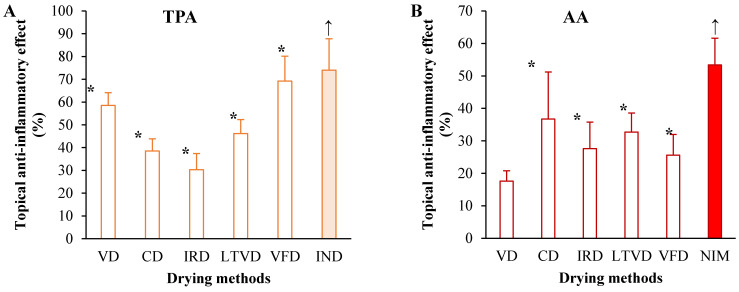
Topical anti-inflammatory effects of extracts reconstituted in acetone, obtained from dried cauliflower by different methods, assessed by mouse ear edema induced by (**A**) phorbol-12-myristate-13-acetate (TPA) and (**B**) arachidonic acid (AA). An asterisk (*) presented on each bar indicates significant differences at *p* < 0.05 between the samples in comparison to the negative control (100% inflammation). Indomethacin (IND) is the reference drug against TPA. Nimesulide (NIM) is the reference drug against AA. ↑ maximum anti-inflammatory effect. Abbreviations: vacuum drying (VD), convective drying (CD), infrared drying (IRD), low-temperature vacuum drying (LTVD), vacuum freeze-drying (VFD).

Cruciferous-derived bioactive compounds, especially GSL breakdown products, have been demonstrated to act as therapeutic agents for inflammatory response [2,47]. Consequently, this study investigated the anti-inflammatory effect of extracts reconstituted in acetone obtained from dried cauliflower by different methods at an animal experimental level by employing two induced mouse ear inflammation models. The following models were applied: (i) 12-O-tetradecanoylphorbol-13-acetate (TPA) is a typical model that induces inflammation by upregulating proinflammatory markers such as interleukins (IL)-1, tumor necrosis factor-α (TNF-α), cyclooxygenase-2 (COX-2) and inducible nitric oxide synthase (i-NOS) [48]; and (ii) arachidonic acid (AA) is also a commonly used animal model that induces rapid inflammation mediated by metabolization of AA through three pathways: via COX-2 that leads to the generation of prostanoids (prostaglandins, prostacyclins and thromboxanes); via lipoxygenase-5 (LOX-5) that results in the generation of leukotrienes and via cytochrome P450 (CYP) to produce eicosanoids [49]. The results showed that the cauliflower extracts (3 mg/ear) had an anti-inflammatory effect by reducing TPA-induced ear edema in mice (Figure 1A).

The extract obtained from cauliflower dried by VFD showed the greater reduction of ear edema (69.2% edema reduction), comparable with the edema-reducing effect of indomethacin (74.0%, positive control), followed by the extracts obtained from VD (58.5% edema reduction) and LTVD (46.2% edema reduction), where the anti-inflammatory effect was also shown clearly. Apparently, the oxygen-deficient environment inside the chamber of each dryer, the application of different sub-atmospheric pressures and the low drying temperatures allow preserving the substantial anti-inflammatory activity of some compounds in cauliflower.

On the other hand, as illustrated in Figure 1B, the extracts obtained from CD and LTVD were more effective against AA-induced edema in mice, where the ear inflammation was reduced to 36.7% and 32.7%, respectively, though it was less effective than nimesulide (53.4%, positive control). In fact, the extract obtained from cauliflower dried by VD did not significantly reduce the skin inflammation induced by AA. These results are consistent with our previous report, in which extracts obtained from broccoli dried by VD were unable to reduce ear edema induced by AA in mice [26].

Overall, the results acquired by these two animal experiments suggested that extracts obtained from cauliflower dried by different methods have anti-inflammatory effects on acute and local inflammations in vivo. The antiedematogenic effect of extracts obtained from cauliflower dried by CD and LTVD might be associated, to a certain extent, with inhibition of COX and LOX pathways, key enzymes of the AA cascade. Aliabadi et al. [50] reported in their review that plant materials such as garlic and quince leaves have potential effects in inhibiting 15-LOX1 and 15-LOX2, while apple and pear leaves have preventive effects on COX-2 activity. Therefore, these materials could be utilized as promising anti-inflammatory agents. Additionally, the extracts obtained from VFD and VD possibly decrease the protein levels of i-NOS and COX-2 and production of inflammatory mediators in the TPA-stimulated mouse ear tissues [51]. However, due to the limitations of the study challenge, further investigations are needed to determine how the underlying molecular mechanisms of the extracts obtained from dried cauliflower exert their anti-inflammatory action on TPA- and AA-induced skin responses in mice.

### 3.6. Antiproliferative Potential in Dried Cauliflower

**Figure 2:**
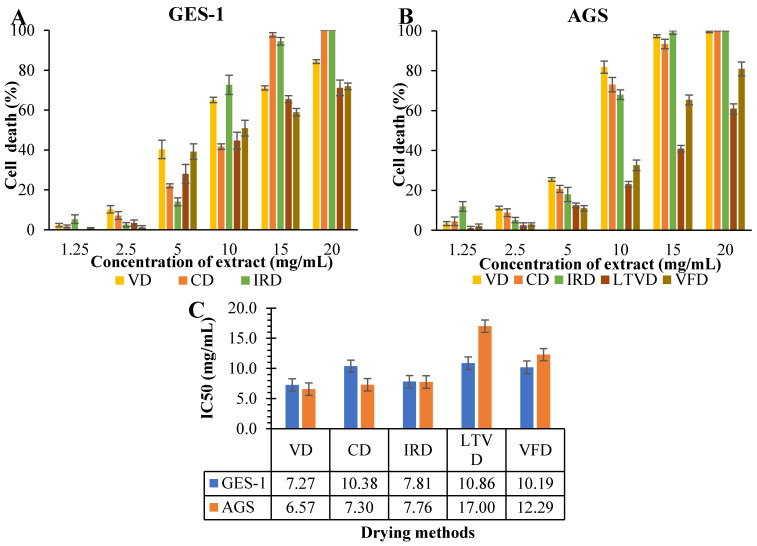
Anti-proliferative effects estimated through the propidium iodide (PI) assay at various concentrations of extracts reconstituted in RPMI medium obtained from cauliflower dried by different methods against (**A**) non-tumorigenic gastric GES-1 cell lines and (**B**) the gastric cancer AGS cell line. (**C**) Estimation of dose required to reduce the number of viable cells by half (IC50). Data are expressed as mean ± SEM of three independent experiments (*n* = 3). Abbreviations: vacuum drying (VD), convective drying (CD), infrared drying (IRD), low-temperature vacuum drying (LTVD), vacuum freeze-drying (VFD).

It is well accepted that the activation of NF-κB pathways can stimulate the expression of various genes with inflammatory activity, such as COX-2 and i-NOS, which can promote the development and progression of gastric cancer [52,53]. In this study, the antiproliferative ability of the extracts, obtained from cauliflower dried by different methods and reconstituted in RPMI medium, against non-tumorigenic gastric GES-1 cells and the gastric cancer AGS cell line was investigated, using serial dilutions of the extracts (20, 15, 10, 5, 2.5, and 1.25 mg/mL) (Figure 2).

As was evident, concentrations at 1.25 and 2.5 mg/mL had no harmful effects on GES-1 (Figure 2A) and AGS (Figure 2B) cells. Instead, at 15 and 20 mg/mL concentration, the extracts obtained from CD and IRD were toxic to both cell lines, with up to 100% cell death after 48 h of treatment. Under the same conditions, the extract obtained from VD also was toxic to AGS cells and was able to reduce about 78% of GES-1 cells on average. This was expected, as some authors have reported that the organic sulfur-containing compounds present in cruciferous vegetables can have cytotoxic effects on both normal and cancerous cells at higher doses [54]. Nonetheless, a natural extract is expected to be highly toxic to malignant cells while causing minimal harm to normal cells. In the present study, extracts obtained from CD and VD seemed be more toxic against cancerous gastric cells (Figure 2B) than healthy gastric cells (Figure 2A) at a concentration of 10 mg/mL. More specifically, this effect can be supported by calculating the dose required to reduce by half the number of viable cells (IC50) in both cell lines. Thus, the extract obtained from VD showed a higher efficiency in reducing by half the number of cancerous AGS cells (IC50 = 6.57 mg/mL), followed by the extract obtained from CD (IC50 = 7.30 mg/mL). However, the same extracts needed higher concentrations (7.27 and 10.38 mg/mL, respectively) to reduce by half the number of healthy GES-1 cells (Figure 2C). Instead, the IRD extract exhibited the same level of toxicity against both cell lines. Interestingly, extracts obtained from LTVD and FD were more cytotoxic toward GES-1 (IC50 = 10.86 and 10.19 mg/mL, respectively) than toward AGS (IC50 = 17.00 and 12.29 mg/mL, respectively) cells, probably due to the higher presence of AITC, which was 26.0 and 18.0 µg/g for LTVD and VFD, respectively (Table 3). This ITC has also been found to exhibit certain toxicity toward normal cells, albeit dependent on the dosage [55], which presents a drawback to its use as an antiproliferative agent.

### 3.7. Neuroprotective Potential in Dried Cauliflower

**Figure 3:**
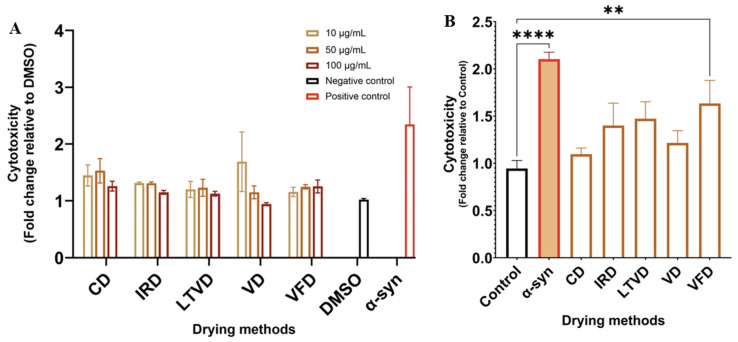
(**A**) Cytotoxicity and (**B**) neuroprotective effects of extracts reconstituted in DMSO obtained from cauliflower dried by different methods: SN4741 cells were treated with cauliflower extract for 24 h and exposed to human α-syn monomers (Mono, control) or α-syn PFF (positive control). DMSO was used as negative control. Data are presented as mean and SEM of three independent experiments performed in triplicate. Statistically significant differences were detected by ordinary one-way ANOVA (**** *p* < 0.0001; ** *p* < 0.01).

Our preliminary studies indicated that extracts from both broccoli [31] and red cabbage [28] obtained from dried material exert neuroprotective effects on mouse dopaminergic neuron SN4741 cells and human neuroblastoma SH-SY5Y cells, respectively. In this study, the protective effect of extracts reconstituted in DMSO obtained from cauliflower dried by different methods on SN4741 neuronal cells using a Parkinson’s disease (PD) model based on α-syn preformed fibril (α-syn PFF) toxicity (Figure 3) was explored.

Firstly, we investigated whether different concentrations of cauliflower extracts triggered a direct cytotoxic effect. The results showed that exposure of SN4741 cells to the amyloid form of α-syn triggered clear cellular toxicity, whereas cauliflower extracts did not exert cytotoxic effects at any concentration compared to the control group (Figure 3A). Consequently, cells were pretreated with cauliflower extracts at a concentration of 50 μg/mL before exposure to α-syn PFF. As observed in Figure 3B, there was a significant reduction in cellular toxicity triggered by α-syn PFF under cauliflower extract treatment. Among the extracts, the extract obtained from CD exhibited the best cytoprotective effect against α-syn PFF toxicity in SN4741 cells, followed by the extract obtained from VD. The cytotoxicity increased when cells were treated with extracts obtained from IRD and LTVD, revealing that both extracts had a lower protective effect on damaged cells compared to the above treatments. Interestingly, treatment with the extract obtained from VFD did not effectively protect cells from damage caused by α-syn PFF, significantly (*p* < 0.01) increasing cytotoxicity (Figure 3B). To better elucidate this behavior, the following section discusses the effects of certain compounds found in the material under study with their anti-inflammatory, antiproliferative and neuroprotective properties.

### 3.8. Relationship between Bioactive Compounds and Health-Related Properties of Cauliflower

**Figure 4:**
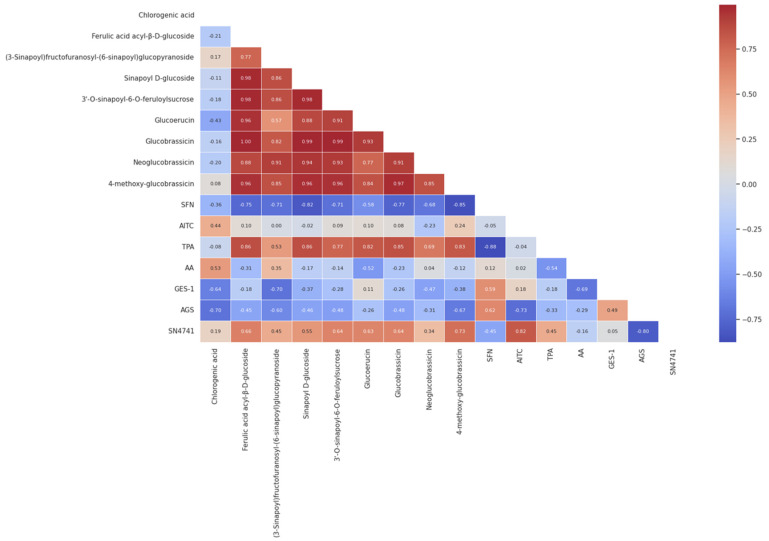
Correlation analysis visualized by heatmap between the bioactive compounds and anti-Inflammatory, antiproliferative and neuroprotective potential of dried cauliflower. Dark red and blue report the degree of significantly positive and negative correlations, respectively.

The importance of detected individual components in dried cauliflower on anti-inflammatory, antiproliferative and neuroprotective properties was confirmed by the Pearson correlation plot, as shown in Figure 4.

The data revealed a strong correlation between some hydroxycinnamic acid derivatives, such as ferulic acid acyl-β-D-glucoside, sinapoyl D-glucoside and 3′-O-sinapoyl-6-O-feruloylsucrose, and an anti-inflammatory effect that was measured on a TPA-induced mouse ear edema model (r = 0.86, 0.86 and 0.77, respectively). As already reported in some previous work on sinapic acid and its derivatives, a relationship was found among these compounds and the inflammation induced in mouse models. In this regard, Yang et al. [56] reported that sinapic acid exhibits anti-inflammatory effects in obesity-induced mice, also reducing the levels of proteobacteria associated with inflammation. Other studies showed that isopentyl sinapate reduced the carrageenan-induced paw edema in rats [57], whereas 1,2-O-disinapoyl glucoside in radish sprouts could alleviate colitis [58]. Meanwhile, Xian et al. [59] reported that sinapine in *Brassica juncea* may act synergistically with GSL (sinalbin) and myrosinase to reduce TPA- and AA-induced ear edema, through down-regulation of the mRNA expression of TNF-α, IL-1β and IL-6. Based on these considerations, we deduced that there is a synergistic effect between cauliflower-derived hydroxycinnamic acid and GSLs (glucoerucin, glucobrassicin, and 4-methoxy-glucobrassicin) in reducing TPA-stimulated ear edema formation, as GSLs were also strongly correlated with this inflammatory model (Figure 4). Contrary to our expectations, a negative correlation was found between the SFN and AITC with the inflammatory model (r = −0.88 and −0.04, respectively). Therefore, additional research is needed to connect each hydroxycinnamic acid and its derivatives, GSLs and ITCs to the production of inflammatory cytokines in in vivo models.

Regarding antiproliferative properties, the results indicated that SFN was positively correlated with the death of non-tumorigenic gastric GES-1 cells (r = 0.59) and the gastric cancer AGS cell line (r = 0.62). It is speculated that, although cauliflower contains many biologically active compounds, the SFN content might be the main reason for its antiproliferative capacity. Previous studies have provided evidence that SFN inhibits cell proliferation and induces apoptosis in gastric cancer cells [60]. This occurs due to mitosis-specific senescence during cell cycle progression through an ROS/AMPK-dependent pathway induced by SFN [61]. However, similarly to AITC [55], elevated concentrations of SFN can also be toxic to non-cancerous cells [62]. The latter could explain the toxic effect of extracts obtained from IRD against both cell lines (Figure 2C) due to the higher content of SFN and AITC in that extract compared to extracts obtained from VD and CD, which had higher SFN content but lower AITC content (Table 3).

Additionally, we investigated the relationship between bioactive compounds and neuroprotective properties of cauliflower extracts. It is important to note that higher cytotoxicity indicates that the extract was less effective in protecting against neuronal cell injury induced by α-syn PFF (Figure 3B). Therefore, the interpretation of Pearson’s correlation coefficients should be considered in the opposite manner. In this scenario, the results indicated that SFN content was mainly correlated with neuroprotective effect. In contrast, AITC content was not correlated with such effect (Figure 4). Consistently, previous studies have shown that AITC does not play a protective role against oxidative stress in astrocytes, whereas SFN was able to counteract ROS production induced by H_2_O_2_ exposure [63]. SFN has also been reported to hinder the progression of Parkinson’s disease through various pathways, as detailed in numerous reviews [64,65,66]. The authors agree that the main molecular mechanisms of action include the modulation of the Nrf2/ARE pathway, inhibition of pro-inflammatory cascades, and stimulation of the neuronal apoptotic pathway. In our study, the cauliflower extracts that contained relatively high amounts of SFN demonstrated a better neuroprotective effect against α-syn PFF toxicity in SN4741 cells (Figure 3B). However, further studies are required to reveal the underlying reasons for the varying neuroprotective effects of each extract obtained from cauliflower subjected to different drying methods.

Finally, it is worth noting that despite the challenges of conducting a human dietary intervention with a food product, there is increasing interest from the pharmaceutical industry in utilizing biocompounds from vegetables, not only as food supplements but also as potential drugs.

## 4. Conclusions

The data from our study indicate that both the VFD and IRD methods can prevent the loss of amino acids involved in GSL anabolism, as well as essential amino acids in cauliflower. Additionally, the VFD method preserved high levels of ferulic acid acyl-β-D-glucoside, (3-sinapoyl)fructofuranosyl-(6-sinapoyl)glucopyranoside, and sinapoyl D-glucoside. The pre-freezing step of samples subjected to VFD could explain the superior retention of GSL content, as enzyme activity was halted. However, this also minimized the transformation of GSLs into SFN. Correlation analysis revealed a strong association between SFN and the antiproliferative and neuroprotective effects of cauliflower extracts, with CD and VD samples exhibiting the highest effects. On the other hand, it is likely that a synergistic effect exists between the derived hydroxycinnamic acid and the GSLs retained in the VFD samples, contributing to the reduction of TPA-stimulated ear edema formation in vivo.

As VFD is the recommended treatment for the preservation of bioactive compounds in cauliflower and the alleviation of induced inflammation in mice, it could be used for pharmaceutical applications and food supplements. However, further research is still needed to gain a more comprehensive understanding of the detailed mechanisms underlying its anti-inflammatory, antiproliferative, and neuroprotective effects. Future efforts should focus on the potential impact of drying on the bioavailability of bioactive compounds in cauliflower

## Figures and Tables

**Table 1:** Effect of five drying methods on amino acid profile of dried cauliflower.

Parameters (g/100 g)	Drying Methods				
	VD	CD	IRD	LTVD	VFD
Essential amino acids (Eaa)					
Histidine	ND	ND	0.23 ± 0.01 ^b^	0.21 ± 0.04 ^b^	0.33 ± 0.03 ^a^
Threonine	0.46 ± 0.01 ^b^	0.10 ± 0.00 ^c^	1.06 ± 0.07 ^a^	0.65 ± 0.07 ^b^	1.07 ± 0.20 ^a^
Valine	0.43 ± 0.02 ^c^	0.31 ± 0.00 ^c^	0.95 ± 0.10 ^a^	0.59 ± 0.17 ^bc^	0.85 ± 0.21 ^ab^
Phenylalanine	0.66 ± 0.08 ^c^	0.51 ± 0.07 ^c^	1.58 ± 0.21 ^a^	1.13 ± 0.10 ^b^	1.45 ± 0.27 ^ab^
Isoleucine	1.11 ± 0.03 ^b^	0.80 ± 0.10 ^b^	1.64 ± 0.18 ^a^	1.06 ± 0.03 ^b^	1.69 ± 0.26 ^a^
Leucine	0.77 ± 0.02 ^a^	0.62 ± 0.11 ^a^	0.75 ± 0.21 ^a^	0.58 ± 0.02 ^a^	0.80 ± 0.01 ^a^
Lysine	0.46 ± 0.01 ^d^	0.26 ± 0.02 ^e^	0.98 ± 0.07 ^b^	0.72 ± 0.07 ^c^	1.33 ± 0.40 ^a^
Total Eaa	3.90 ± 0.18	2.59 ± 0.30	7.21 ± 0.84	4.94 ± 0.49	7.53 ± 1.39
Nonessential amino acids (NEaa)					
Aspartic acid	0.86 ± 0.02 ^bc^	0.56 ± 0.07 ^c^	1.48 ± 0.16 ^a^	0.68 ± 0.22 ^c^	1.35 ± 0.36 ^ab^
Glutamic acid	1.53 ± 0.00 ^bc^	1.00 ± 0.13 ^c^	2.25 ± 0.27 ^ab^	1.57 ± 0.12 ^bc^	2.53 ± 0.71 ^a^
Serine	0.52 ± 0.21 ^b^	0.55 ± 0.06 ^b^	0.96 ± 0.12 ^a^	0.69 ± 0.04 ^ab^	0.97 ± 0.19 ^a^
Glycine	0.93 ± 0.02 ^a^	1.03 ± 0.15 ^a^	0.63 ± 0.12 ^bc^	0.56 ± 0.11 ^c^	0.86 ± 0.01 ^ab^
Arginine	0.77 ± 0.04 ^ab^	0.56 ± 0.06 ^c^	0.84 ± 0.10 ^a^	0.64 ± 0.05 ^bc^	0.87 ± 0.05 ^a^
Alanine	0.63 ± 0.02 ^bc^	0.50 ± 0.06 ^c^	0.96 ± 0.12 ^a^	0.67 ± 0.05 ^bc^	0.81 ± 0.16 ^ab^
Tyrosine	0.32 ± 0.04 ^a^	0.36 ± 0.06 ^a^	0.41 ± 0.01 ^a^	0.24 ± 0.05 ^a^	0.43 ± 0.07 ^a^
Total NEaa	5.56 ± 0.34	4.56 ± 0.59	7.54 ± 0.91	5.05 ± 0.64	7.81 ± 1.55
Total aa	9.46 ± 0.52	7.15 ± 0.89	14.75 ± 1.75	9.99 ± 1.13	15.34 ± 2.94

Values are expressed as mean ± standard deviation (*n* = 3). Different alphabet letters presented in the same row indicate significant differences at *p* < 0.05 among different drying methods. Abbreviations: Not detected (ND), vacuum drying (VD), convective drying (CD), infrared drying (IRD), low-temperature vacuum drying (LTVD), vacuum freeze-drying (VFD).

**Table 2:** Effect of five drying methods on hydroxycinnamic acid and its derivatives and glucosinolate profile (µg/g dried sample) of dried cauliflower.

N°	Identification	[M-H]-Theoretical *m*/*z*	[M-H]-Observed *m*/*z*	Mass Error (ppm)	Drying Methods				
					VD	CD	IRD	LTVD	VFD
1	Chlorogenic acid	353.0878	353.0876	−0.56	2.63 ± 0.37 ^d^	14.05 ± 0.64 ^b^	1.33 ± 0.43 ^d^	48.35 ± 2.67 ^a^	7.47 ± 0.46 ^c^
2	Ferulic acid acyl-β-D-glucoside	355.1035	355.1043	2.25	12.49 ± 0.41 ^b^	4.67 ± 0.15 ^c^	0.85 ± 0.05 ^d^	6.12 ± 0.42 ^c^	51.79 ± 2.57 ^a^
3	(3-Sinapoyl)fructofuranosyl-(6-sinapoyl)glucopyranoside	753.2247	753.2267	2.65	18.01 ± 0.68 ^d^	32.88 ± 2.41 ^b^	18.24 ± 2.14 ^d^	28.35 ± 0.58 ^c^	44.17 ± 4.20 ^a^
4	Sinapoyl D-glucoside	385.1140	385.1154	3.64	125.91 ± 6.10 ^b^	125.05 ± 11.68 ^b^	35.34 ± 1.27 ^c^	108.62 ± 8.05 ^b^	344.14 ± 17.81 ^a^
5	3′-O-sinapoyl-6-O-feruloylsucrose	723.2141	723.2185	6.08	9.18 ± 0.65 ^cb^	11.13 ± 0.48 ^b^	6.46 ± 0.44 ^c^	9.00 ± 0.35 ^bc^	37.06 ± 4.27 ^a^
6	Glucoerucin	420.0351	420.0371	4.76	18.47 ± 1.18 ^c^	ND ^d^	8.31 ± 0.74 ^b^	ND ^d^	50.24 ± 1.32 ^a^
7	Glucobrassicin	447.0537	447.0556	4.25	1034.83 ± 35.03 ^b^	863.83 ± 39.63 ^c^	502.57 ± 23.56 ^d^	880.62 ± 23.89 ^c^	2950.36 ± 166.4 ^a^
8	Neoglucobrassicin	477.0643	447.0648	1.05	703.55 ± 51.07 ^c^	1412.46 ± 84.09 ^b^	251.43 ± 20.99 ^e^	516.58 ± 25.15 ^d^	2520.91 ± 109.4 ^a^
9	4-methoxy-glucobrassicin	477.0643	447.0688	9.43	243.88 ± 15.63 ^c^	225.47 ± 24.15 ^c^	145.32 ± 7.79 ^d^	340.19 ± 16.01 ^b^	693.76 ± 18.81 ^a^

Values are expressed as mean ± standard deviation (*n* = 3). Different alphabet letters presented in same row indicate significant differences at *p* < 0.05 among different drying methods. [M-H]⁻ indicates a molecular ion that has lost a proton (H⁺), resulting in a negative charge and a reduction in mass by 1.0073 Da. Theoretical *m*/*z* is the value expected to be observed in the mass spectrum based on the molecular formula and the changes that occur during ionization (such as the loss or gain of protons). Observed *m*/*z* is the *m*/*z* value recorded in the experimental mass spectrum for that specific ion. Mass error: Errors greater than 20 ppm could indicate that another molecule with a similar mass has been detected. Abbreviations: Not detected (ND), mass-to-charge (*m*/*z*), vacuum drying (VD), convective drying (CD), infrared drying (IRD), low-temperature vacuum drying (LTVD), vacuum freeze-drying (VFD).

**Table 3:** Effects of five drying methods on isothiocyanate content (µg/g dried sample) of dried cauliflower.

Parameters	Drying Methods				
	VD	CD	IRD	LTVD	VFD
SFN	8.00 ± 0.10 ^c^	9.00 ± 0.10 ^b^	12.04 ± 0.10 ^a^	7.00 ± 0.10 ^d^	5.40 ± 0.10 ^e^
AITC	6.00 ± 0.30 ^c^	ND	22.02 ± 3.00 ^ab^	26.02 ± 3.00 ^a^	18.01 ± 1.30 ^b^
PITC	ND	ND	ND	ND	ND
I3C	ND	ND	ND	ND	ND

Values are expressed as mean ± standard deviation (*n* = 3). Different alphabet letters presented in same row indicate significant differences at *p* < 0.05 among different drying methods. Abbreviations: Not detected (ND), sulforaphane (SFN), allyl isothiocyanate (AITC), phenyl isothiocyanate (PITC), indole-3-carbinol (I3C), vacuum drying (VD), convective drying (CD), infrared drying (IRD), low-temperature vacuum drying (LTVD), vacuum freeze-drying (VFD).

## Data Availability

The datasets produced in this study are available upon request from the corresponding author.
